# Lost highway(s): barriers to postnatal cortical neurogenesis and implications for brain repair

**DOI:** 10.3389/fncel.2015.00216

**Published:** 2015-06-16

**Authors:** Aslam Abbasi Akhtar, Joshua J. Breunig

**Affiliations:** ^1^Board of Governors Regenerative Medicine Institute, Cedars-Sinai Medical CenterLos Angeles, CA, USA; ^2^Department of Biomedical Sciences, Cedars-Sinai Medical CenterLos Angeles, CA, USA; ^3^Samuel Oschin Comprehensive Cancer Institute, Cedars-Sinai Medical CenterLos Angeles, CA, USA

**Keywords:** reprogramming, transdifferentiation, regeneration, neurogenesis, brain repair, ventricular zone, gliogenesis

## Abstract

The genesis of the cerebral cortex is a highly complex and tightly-orchestrated process of cell division, migration, maturation, and integration. Developmental missteps often have catastrophic consequences on cortical function. Further, the cerebral cortex, in which neurogenesis takes place almost exclusively prenatally, has a very poor capacity for replacement of neurons lost to injury or disease. A multitude of factors underlie this deficit, including the depletion of radial glia, the gliogenic switch which mitigates continued neurogenesis, diminished neuronal migratory streams, and inflammatory processes associated with disease. Despite this, there are glimmers of hope that new approaches may allow for more significant cortical repair. Herein, we review corticogenesis from the context of regeneration and detail the strategies to promote neurogenesis, including interneuron transplants and glial reprogramming. Such strategies circumvent the “lost highways” which are critical for cortical development but are absent in the adult. These new approaches may provide for the possibility of meaningful clinical regeneration of elements of cortical circuitry lost to trauma and disease.

## Introduction

Comprising 10–26 billion neurons and a similar number of glia (Azevedo et al., 2009; Herculano-Houzel, 2009), and possessing roughly a trillion synapses per cubic centimeter (Drachman, 2005), the mammalian cerebral cortex is one of the most complex structures known to man. Through tightly orchestrated interactions between these neurons and glia, along with communication to and from other brain regions, the cortex mediates higher-order cognition, coordinates motor function, visual perception, somatosensory perception, memory, and a host of other processes (Shipp, 2007). Because of this complexity, developmental disorders often lead to lifelong impairment or disability (Francis et al., 2006). Further, trauma or disease resulting in neurodegeneration in the cortex almost inevitably leads to impairment of psychosocial, cognitive, motor, visual or somatosensory function, depending on the age of the individual and site(s) of neuronal loss (Van Hoesen et al., 1991; Johansson, 2000; Rosema et al., 2015).

To compound this, the primate cortex has almost no proclivity for de novo neurogenesis after birth (Rakic, 1985, 2002a,c; Kornack and Rakic, 2001; Spalding et al., 2005; Bhardwaj et al., 2006). The evidence for and against postnatal neurogenesis in the cortex of primates and other mammals has been extensively reviewed elsewhere and less recent findings will not be discussed here (Rakic, 2002b; Breunig et al., 2007, 2011; Feliciano and Bordey, 2013). Nevertheless, despite the contradictory data from model organisms, carbon-14 dating of human cortical neurons under normal and post stroke conditions indicates that these cells are born prenatally (Huttner et al., 2014), though adult neurogenesis was detected with similar methods in the human hippocampus where it had been previously observed (Eriksson et al., 1998; Spalding et al., 2013). Therefore, cortical neurogenesis is unlikely to significantly contribute to plasticity after cortical injury.

Taken together, directed regeneration of cortical circuitry, however difficult the prospect may be, is one of the few approaches available to potentially ameliorate functional deficits due to cortical neurodegeneration. Though, it is possible that functional recovery after neurodegeneration may turn out to be a matter of generating the appropriate cell numbers and types; developmental cortical disorders such as those seen in *Reeler* mutants—where neurons are generated normally but migrate inappropriately, resulting in mental retardation—suggests that a more meticulous reconstruction of circuitry, one that mimics the pattern of normal development may be necessary (Caviness and Rakic, 1978). Here we review cortical development in this context and describe emerging approaches to generate cortical neurons for cell therapy or *in situ* replacement.

## Cortical Development

**Figure 1:**
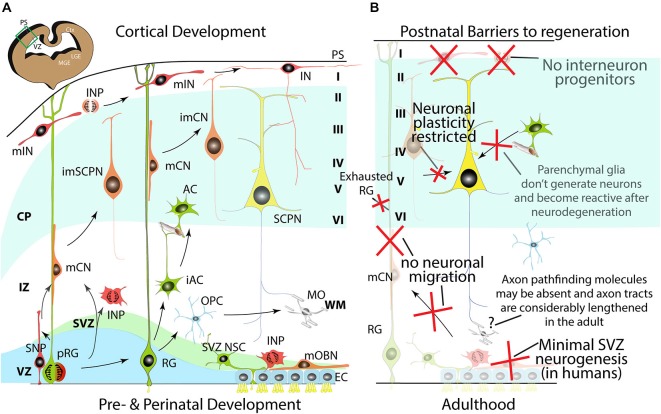
**Cortical development and barriers to regeneration**. **(A)** Schematic representation of neocortical development from neurogenesis through postnatal stages. Approximate location denoted by green box in coronal brain. Proliferative radial glia (pRG) asymmetrically divide to generate intermediate progenitors (INPs) and short neural precursors (SNPs) which proliferate and contribute to the migratory cortical neuron (mCN) population. This population migrates along radial glia and into the cortical plate (CP), where they exit the RG fiber and begin the maturation process. As neurogenesis procedes, progressively more superficial layers are generated (i.e., Layer VI, then Layer V, etc.) Depicted here is the generation of Layer V subcortical projection neurons (SCPN) and the later generation of an upper layer immature callosal neuron (imCN). (Other layers are not depicted for the sake of space and clarity). In parallel, migrating interneurons (mINs) from the ventral telencephalon invade and can proliferate locally prior to maturation into functional interneurons (INs). After neurogenesis ceases, gliogenesis commences en force with the conversion of some RG into immature and subsequently mature astrocytes (iACs and ACs, respectively). Also, oligodendrocyte progenitor cells (OPCs) are born and differentiate into myelinating oligodendrocytes (MOs) in the white matter (WM). Other RG transition into subventricular zone neural stem cells (NSCs), and ependymal cells (ECs). NSCs can give rise to INPs, which generate migrating olfactory bulb-destined neurons (mOBNs). **(B)** In the postnatal and adult brain, significant barriers to regeneration are present. Radial glia are exhausted and become a “lost highway” to any neuronal migration. Similarly, cortical neurons are no longer generated and thus virtually no neurons can be found migrating into the cortex. Neuronal plasticity becomes significantly attenuated, preventing the type of plasticity observed prior to developmental critical periods. Interneuron progenitors and mINs disappear. Parenchymal glia do not cross lineage boundaries and become reactive after injury and degeneration. Moreover, the axon lengths become many fold longer in the adult due to the growth of the organism. For example, a SCPN may reach almost a meter in length while the initial axon started at a few millimeters before progressive lengthening. Finally, in humans there is minimal subventricular zone (SVZ) neurogenesis in the adult, inhibiting strategies which might utilize such cells. Abbreviations: PS, pial surface; IZ, intermediate zone; MGE, medial ganglionic eminence; LGE, lateral ganglionic eminence; Ctx, cortex.

The majority of cortical neurons and glia arise from radial glia stem cells which reside along the ventricle (Figure 1A) and provide highway-like migratory substrates from the ventricle to the pial surface (Rakic, 1972; Noctor et al., 2001; Breunig et al., 2011). During neurogenesis, radial glia divide asymmetrically to generate developing neurons which migrate along these scaffolds and exit off into their respective cortical layers (Figure 1A; Noctor et al., 2001; Rakic, 2009). The cortex is distinctly organized into six horizontal layers which are generated embryonically in an inside-out fashion (Rakic, 2009). As development progresses, generation of lower layers tapers off into genesis of progressively more superficial layers due to an underlying transcriptional program (Kwan et al., 2012). As neurogenesis completes, the “gliogenic switch” shifts the fate of radial glial stem cells towards the genesis of astrocytes and oligodendrocyte progenitor cells (Figure 1A; Rowitch and Kriegstein, 2010). Hence, the majority of cortical neurogenesis occurs prior to birth, while gliogenesis is a perinatal and postnatal phenomenon (Bhardwaj et al., 2006; Yeung et al., 2014).

The majority of neurons in the cortex are glutamatergic while the remaining (roughly 20%) of neurons are GABAergic interneurons (Chu and Anderson, 2015). These interneurons are generated in the subcortical areas of the ventral telencephalon in the rostral forebrain, specifically in the medial ganglionic eminence (MGE) and caudal ganglionic eminence (Anderson et al., 2002; Nery et al., 2002; Wonders and Anderson, 2006; Southwell et al., 2014). Immature interneurons migrate to the developing and neonatal cortex, attain their mature fate (Figure 1A), and integrate their circuitry wherein they function to modulate neuronal activity through inhibitory neurotransmission (Nery et al., 2002; Wonders and Anderson, 2006; Hansen et al., 2013). In humans, evidence exists for the local generation of interneurons in the cortical ventricular and subventricular zone (VZ and SVZ), respectively during embryogenesis, but this remains contentious (Letinic et al., 2002; Hansen et al., 2013; Radonjić et al., 2014). Moreover, there is evidence that interneuron progenitors can proliferate extensively during their migration, including in the cortex and, in addition, the tail end of this migrating progenitor population can be observed postnatally (Costa et al., 2007; Inta et al., 2008; Breunig et al., 2012; Levy et al., 2014).

Due to the intricate spatial and temporal cues that orchestrate cortical development, the task of regenerating the cortex in adulthood possesses many challenges (Figure 1B). Physically speaking, projection neurons in the cortex reach their respective targets early in development (e.g., the spinal cord for Layer V neurons and the thalamus for Layer VI neurons). After axonal targeting is achieved in embryogenesis, these processes progressively lengthen with age from a distance of millimeters and centimeters to almost a meter for an adult corticospinal projection neuron. Furthermore, the cortical radial glia population is exhausted in early postnatal life leaving no scaffold for migration to respective cortical layers (Tramontin et al., 2003). The absence of a path to cortical layers from the ventricular zone represents a significant “lost highway” that may impede regeneration (Figure 1B). Further, neurons are postmitotic cells that do not divide once they have reached their final position in the cortex. And though precursors do exist around the lateral ventricles in the human, akin to development, they largely lose their neurogenic ability *in vivo* (Figure 1B; Sanai et al., 2011; discussed in detail below).

## Postnatal Forebrain Cell Genesis

The neurogenic niches of the adult brain have received a great deal of attention over the past fifteen years due to their ability to contribute to brain plasticity and serve as a model and perhaps substrate for regeneration. The two neurogenic niches in the adult mammalian brain are the subventricular zone (SVZ) of the lateral ventricles and the subgranular zone (SGZ) of the hippocampus (Song et al., 2002; Alvarez-Buylla and Lim, 2004; Ming and Song, 2011). The SVZ neural stem cells are derived from embryonic and perinatal radial glia (Merkle et al., 2004). As these radial glia transition into neural stem cells of the astrocyte lineage, they lose the long basal process that they possess (and therefore the migratory substrate for neurons to migrate into the parenchyma—the eponymous “lost highway”). These Gfap^+^ neural precursors also serve to populate the cortex and striatum with astrocytes (Ge et al., 2012) and oligodendrocytes (Menn et al., 2006), as well as seed the olfactory bulb (OB) with neurons (Alvarez-Buylla and Lim, 2004; Ming and Song, 2011). Specifically, SVZ precursors generate transit amplifying cells which proliferate and generate neuroblasts (Doetsch et al., 1999). These neuroblasts migrate in a clustered, tangential fashion to the OB through the rostral migratory stream (RMS), a structure of ensheathing glial fibers and migrating neurons between the lateral ventricle and OB core (Lois et al., 1996). Upon reaching the OB core, the immature neurons migrate radially towards the OB periphery, establish synapses, and mature into OB interneurons (Carleton et al., 2003; Ming and Song, 2011). Importantly, OB neurogenesis does not appear to be lifelong in humans and instead may be supplanted by striatal interneuron genesis, which does not appear to occur in other non-human primates and rodents (Ernst et al., 2014). Unlike neural precursors, the glial subtypes generated by SVZ radial glia extensively divide perinatally throughout the forebrain (Ge et al., 2012; Yeung et al., 2014). Astrocyte turnover attenuates perinatally in rodents, but polydendrocytes of the oligodendrocyte lineage are believed to be the most proliferative cell type in the adult brain (Burns et al., 2009; Geha et al., 2010).

In the postnatal hippocampal SGZ, radial glia-like neural stem cells give rise to intermediate progenitors (INPs) which in turn generate neuroblasts (Seri et al., 2004). These neuroblasts migrate to the inner granule cell layer and differentiate into granule cells which project to the CA3 region of the hippocampus (Ming and Song, 2011). Several studies in mice have associated changes in hippocampal neurogenesis to learning and memory (Deng et al., 2009, 2010; Mu and Gage, 2011) though a new genetically modified rat allowing for ablation of adult neurogenesis failed to find a similar correlation (Groves et al., 2013). Importantly, evidence of human hippocampal neurogenesis is observed late into life (Eriksson et al., 1998; Spalding et al., 2013). Taken together, the adult brain does have active sites of neurogenesis, but postnatal cortical neurogenesis has seemingly been selected against by evolution. Nevertheless, these neurogenic zones have provided insight into the mechanisms of postnatal neurogenesis and have also served as experimental testing platforms for various genetic experiments which aim at using stem cells for postnatal cortical neurogenesis. In particular, the postnatal cortical SVZ is of increased interest due to its proximity to the cortex. Furthermore, in addition to techniques such as viral-mediated transgenesis, which can manipulate progenitors in the SVZ and SGZ (Braun et al., 2013; Zuccotti et al., 2014); novel techniques such as electroporation allow rapid transgenic manipulation of SVZ progenitors pre- and postnatally, without the need of virus generation (Breunig et al., 2007).

## Learning from Development

In light of the radial glia-to-cortical neuron development paradigm which exists embryonically, neuroscientists have strived to achieve adult cortical neurogenesis from several angles, namely: (1) the redevelopment of radial glia to serve as substrates for differentiation to cortical neurons or as scaffolds for nascent neurons generated through other means; (2) the transplantation of neural stem and progenitor cells to the cortex; and (3) the analysis of developmental genetic factors which direct stem and progenitor cells to become cortical neurons, and as such, the effect of their misexpression in astrocytes and neurons. Below we detail examples of the work neuroscientists have achieved which show glimpses of postnatal neurogenesis in a region of the brain that was initially thought to be cemented during early development. Due to the numerous reviews present in this field, we will focus on recent findings in respect to cortical regeneration and direct the reader towards other reviews for an in-depth analysis of postnatal neurogenesis (Breunig et al., 2007; Urbán and Guillemot, 2014).

### Promoting Radial Glia Reemergence and Alternate Migratory Substrates

Radial glia are necessary for the development of the cortex as they divide asymmetrically to generate immature neurons and are scaffolds for migrating neurons as they travel to their respective layers (Rakic, 2003; Breunig et al., 2011). Efforts have been made to generate radial glia in adulthood to serve as: (1) a source of progenitors for replacing cortical neurons; and (2) scaffolds for migration of neurons into the cortex. In this regard, expression of the tyrosine kinase receptor, ErbB2, in cortical quiescent mature astrocytes for 3 weeks enabled a subset of these astrocytes to assume a radial glia-like phenotype (Ghashghaei et al., 2007). In addition to possessing elongated processes, these “induced radial glia” had increased expression of the Notch 1 ligand which has been shown to be essential for the maintenance of radial glia identity. Other transcription factors required for radial glial maintenance, Sox2, Pax6, and Hes5 (the downstream targets of Notch1) were also upregulated in the induced cells. Interestingly, only astrocytes surrounding the ventricles, but not those in the cortical parenchyma, were able to re-assume their radial glia identity, suggesting that not all astrocytes in the cortex are the same. Induced radial glia were able to give rise to new neurons *in vitro* and *in vivo*, and also supported the migration of transplanted embryonic-day 16 cortical neurons. Similar work was done by Gregg and Weiss (2003) where epidermal growth factor (EGF) expression in cultures of forebrain neural stem cells resulted in an adoption of radial glia morphology, expression of Nestin and RC2, and supported the migration of immature neurons. Infusion of EGF into the adult forebrain lateral ventricle also led to the generation of radial glia-like cells within the adult forebrain ependyma (Gregg and Weiss, 2003). In addition, the endocytic adaptor proteins Numb and Numbl have been identified as regulators of radial glia adhesion and polarity (Rasin et al., 2007). Though originally identified in Drosophila neural progenitors for its role in promoting neuronal cell fate by inhibiting notch signaling, when Numb and Numbl were inactivated in mouse radial glia, adheren junctions and polarity were disrupted through disrupted cadherin trafficking, resulting in progenitor cell dispersion and disordered cortical lamination (Rasin et al., 2007). In contrast, when Numb or cadherin was overexpressed, radial glia were maintained postnatally beyond the standard neurogenic period in the cortex (Rasin et al., 2007).

Investigation into the migrational modalities used by OB neurons may also provide insight regarding different migratory substrates that may potentially be utilized for induced cortical neurogenesis. As previously mentioned, OB neurons are one of the few neuronal populations continuously generated in the adult rodent. These nascent neurons migrate radially from the OB core to the OB periphery. However, the OB does not contain radial glia-like projections or an RMS-like glial sheath to serve as scaffolds for these migrating neurons. Upon investigating the mode of travel of these neurons, it was found that these nascent interneurons use the OB vasculature as a scaffold for migration (Bovetti et al., 2007; Tanaka et al., 2011). This new modality, termed *vasophilic migration* is facilitated through an interaction between the extracellular matrix and perivascular astrocyte end feet (Bovetti et al., 2007). Therefore, it is possible that vasculature in the cortex may serve as a modality for migration of nascent neuronal populations to their respective cortical layers. Indeed, there is evidence that upon focal microlesions, SVZ cells can generate neurons in the cortex (Magavi et al., 2000; Brill et al., 2009) but more recent work failed to find new neurons after employing two different genetic lesion paradigms (Diaz et al., 2013).

### Employing Developmental Lessons to Facilitate Transplantation

The creation of induced pluripotent stem cells (iPSCs), which led to Shinya Yamanaka receiving the 2012 Nobel Prize, energized the field of regenerative medicine in several ways. First, the finding that pluripotency can be induced in postmitotic cell types opens the door to myriad possibilities in terms of reprogramming and directed differentiation. Secondly, iPSCs now provide a virtually unlimited source of patient-matched cells for transplantation. Over the last two decades, numerous groups have generated various cortical neuronal subtypes from embryonic and induced pluripotent stem cells *in vitro* (Gaspard et al., 2008; Hansen et al., 2011; Shi et al., 2012; Espuny-Camacho et al., 2013; Michelsen et al., 2015). In parallel with these findings, investigators have also demonstrated that a three-dimensional culture of pluripotent stem cells can “self-organize” into a complex tissue with striking similarities to the cerebral cortex, suggesting that neural stem cells may possess the inherent cues needed for cortical formation (Lancaster et al., 2013). This raises the prospect of transplanted stem cells to replace damaged or diseased neurons in the adult cortex. This possibility would exist *if* the adult cortex maintained the intrinsic *spatial* cues of development in order to direct the differentiation of *temporally* relevant stem cells. In this regard, (Ideguchi et al., 2010) derived mouse embryonic stem (ES) cells from the E4.5 blastocyst and differentiated them for 7 days before transplanting into various regions of the cortex. The cells exhibited region specific projections, with cells transplanted to the motor or visual cortex projecting to their respective targets. In a similar experiment, (Gaspard et al., 2008) transplanted cells were differentiated for 12–17 days (instead of 7) and did not present the region specific projections as all cells projected to visual cortex targets, despite being transplanted to the frontal cortex. This suggests that a variation of 5–10 days of *in vitro* differentiation plays a role in the success of graft integration and specificity, and highlights the need for *temporal* specificity of the transplanted cells. Furthermore, recent work further illustrates the need for matching the areal identity of transplanted neurons (Michelsen et al., 2015). In this study, visual cortex neurons were differentiated from mouse ES cells *in vitro* for 14 days and transplanted into the adult visual cortex after a focal neurotoxic lesion. The transplanted cells integrated into their respective pathways and electrophysiological recordings revealed the cells were responsive to visual stimuli. Similar to previous studies, significant integration and engraftment were not observed when ES cell-derived visual cortex neurons were transplanted into the motor cortex, or when motor cortex neurons were transplanted into the visual cortex, indicating the need for *areal* specificity. Importantly, recent work suggests that *in vitro* differentiation of ES-derived neural progenitors towards cortical fates fails to properly recapitulate *in vivo* development in many aspects, including aberrant progenitor specification and stalled differentiation (Sadegh and Macklis, 2014). It should be noted that this report (Sadegh and Macklis, 2014) used mouse ES cells cultured in a monolayer and allowed to mature in a manner previously used for cortical differentiation. Taken together, these findings suggest that neuronal replacement using pluripotent stem cells combined with specific differentiation protocols holds promise. However, they also suggest that it will be important to spatiotemporally match donor cells for transplantation and this will require a requisite understanding of the mechanisms of areal identity in order to specify the appropriate populations from pluripotent cells. In this regard, much work remains to be done to understand how to appropriately derive specific cortical cells and tissues from pluripotent cell types despite an increasing understanding of how this process occurs during development (Rakic et al., 2009; Kwan et al., 2012; Greig et al., 2013). Clinically, the long-term safety issues of iPSC-based cell therapy will need more thorough evaluation in light of recent findings of oncogenic transformation of iPSC-derived NSCs when transplanted into the spinal cord (Nori et al., 2015). However, the iPSCs from this study were generated using lentivirus (Nori et al., 2015) and it remains to be seen if similar problems will emerge from non-integrating iPSC generation methods where continued transgene mis-expression is unlikely. In the interim, the use of iPSCs for generating neurons for disease modeling and drug screening will likely be of great importance.

### Interneuron Transplantation

As previously mentioned, unlike other neuronal subtypes in the cortex which are generated locally from radial glia, interneurons are generated from the ganglionic eminences of the ventral telencephalon and migrate to the cortex (among other regions of the brain) where they mature and integrate into their respective networks (Noctor et al., 2004; Southwell et al., 2014). While projection neurons of the cortex target various intra- and extra-cortical regions, cortical interneurons project onto other neurons in the cortex and inhibit neurotransmission within this region. This balance of inhibitory/excitatory signals is critical for the proper function of the cortex. Conditions such as epilepsy and schizophrenia are thought to be related at least in part to dysfunctional interneurons, giving them the title of “interneuronopathies” (Kato and Dobyns, 2005; Southwell et al., 2014).

As cortical interneurons exhibit a developmental paradigm which involves long distance migration from extra-cortical regions followed by maturation and integration in the cortex, the use of interneuron progenitors in transplant therapy for interneuron replacement may be more feasible compared to transplant efforts aimed at replacing cortical projection neurons. For a detailed review of interneuron transplants, we direct the reader to Alvarez Dolado and Broccoli (2011) and Southwell et al. (2014). Indeed, interneurons do not require radial glia-like scaffolds to migrate from the ganglionic eminences to the cortex, and the neuronal subtypes they project on are not as distant as the respective targets of projection neurons. To this, several groups have achieved promising results transplanting interneurons generated from the embryonic MGE, ES cells, and iPSCs (Tanaka et al., 2011; Maroof et al., 2013; Nicholas et al., 2013; Southwell et al., 2014). Transplanted interneurons migrate from the graft site throughout the cortex and successfully integrate into the young and adult rodent cortex (Maroof et al., 2013; Nicholas et al., 2013). Unlike the transplantation of other cell types which has resulted in decreased transplant survival or a decrease in the native population, which may be detrimental, transplanted interneurons increase the overall pool of cortical interneurons (Southwell et al., 2010). Notably, the cortex has a limit of how many interneurons it can support, which is reported to be roughly 10% more than the native population (Southwell et al., 2010, 2014). Transplanted interneurons display spontaneous and induced synaptic currents and further, transplantation has been shown to increase inhibitory signals in glutamatergic neurons (Southwell et al., 2010; Bráz et al., 2012). Additionally, several groups have reported functional benefits from cortical interneuron transplants in mouse models of epilepsy and schizophrenia (Baraban et al., 2009; Tanaka et al., 2011). Specifically, (Baraban et al., 2009) transplanted precursor cells from the embryonic E13.5 MGE into the postnatal day 2 mouse brain and reported their maturation into GABAergic interneurons when analyzed 30 days after transplantation. The transplanted cells integrated and dispersed throughout the cortex as indicated by immunohistochemistry, electron microscopy, electrophysiology, and increased GABA-mediated synaptic inhibition on pyramidal neurons. Bilateral grafts of the embryonic MGE cells into experimental epileptic mice reduced the duration and frequency of spontaneous electrographic seizures. (Tanaka et al., 2011) transplanted MGE cells of the same embryonic age (E13.5) into the medial prefrontal cortex and observed similar functional integration as well prevention of phencyclidine-induced cognitive defects.

While diseases that independently affect cortical projection neurons may not directly benefit from the increased inhibition provided by transplanted interneurons, these migratory neurons may be used as a vehicle for delivery of trophic factors. Traditionally, astrocytes have been used in this regard as they have been viewed as a resilient supportive cell type for neurons. Among the characteristics that have made astrocytes more appealing are their ability to divide after transplantation, migrate to sites of injury, ensheathe neurons, and their ease of generation *in vitro* (Svendsen et al., 1997; Behrstock et al., 2006, 2008; Suzuki et al., 2007). However, recent reports of successful interneuron generation from iPSCs, engraftment after transplantation, and transgenic manipulation may suggest interneurons as tools for factor delivery to areas of the cortex (Southwell et al., 2014).

## Genetic Engineering and Reprogramming

**Figure 2:**
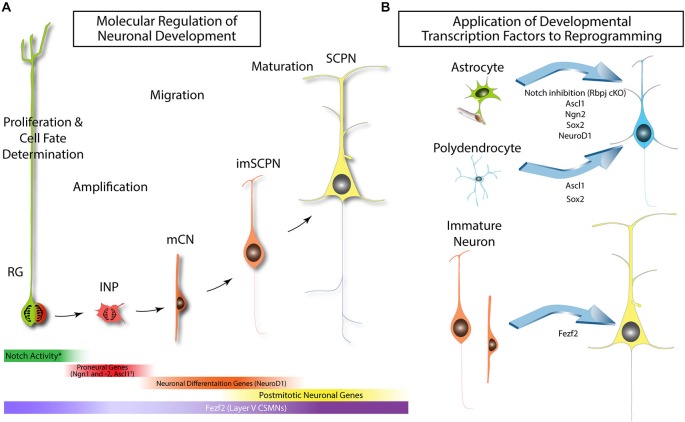
**Molecular control of neurogenesis and its use in reprogramming**. **(A)** Radial glia utilize Notch signaling to self-renew during mitosis. (*-during asymmetric division the cell inheriting the basal process exhibits high Notch activity while the other daughter displays diminished Notch activity). Neuronal daughter cells upregulate proneural genes such as Ngn1 and Ngn2 in the cortex. (^†^-Ascl1 is the predominant proneural gene in the ventral telencephalon among other regions). Basic helix-loop-helix (bhlh) transcription factors such as NeuroD1 regulate the terminal differentiation of migrating neurons into mature projection neurons. Fezf2 cooperates with all of these factors in the specification of subcortical projection neurons (SCPNs), leading to the eventual expression of a diverse array of transcription factors involved in the postmitotic identity of this neuronal subtype. **(B)** Transgenic misexpression strategies for reprogramming of disparate cell types to neurons.

Over the last decade, “direct reprogramming” or “transdifferentiation” has allowed for terminally differentiated cells to directly assume fate of another differentiated cell type without having to go through a state of pluripotency. Direct reprogramming of neurons from a host of different terminally differentiated cells such as fibroblasts, pericytes, hepatocytes, and other neural cells has been described (Heinrich et al., 2010, 2014; Vierbuchen et al., 2010; Marro et al., 2011; Karow et al., 2012). Much of the discovery and success of these direct reprogramming experiments have come to fruition due to our increasing knowledge of the molecular control of neurogenesis and the advances in transcriptomics over the last decade. For example, neurogenesis follows a general pattern (Figure 2A) whereby a neuronal progenitor is generated by Notch-mediated asymmetric cell division of a neural stem cell (Ables et al., 2011). Among the resulting pair of cells after mitosis, the Notch^High^ cell will remain a stem cell and the Notch^Low^ cell will subsequently upregulate proneural genes such as *Neurog1* and *Neurog2* (or *Ascl1* in other regions) and become an intermediate progenitor (Figure 2A; Bertrand et al., 2002; Ables et al., 2011). This progenitor will continue to terminally differentiatiate under the regulation of basic helix-loop-helix transcription factors such as NeuroD1 (Figure 2A) until later maturation (Guillemot, 2007). Finally, any number of transcription factors involved in neuronal subtype specification will be expressed to control neuronal identity (Kwan et al., 2012). Transcriptomics has led to the discovery of several “master regulators” which specify particular neuronal subtypes (Kwan et al., 2012; Greig et al., 2013) such as Fezf2 (discussed in detail below). As mature cortical projection neurons cannot be transplanted, the ability to directly differentiate or reprogram other cell types into precise neuronal subtypes with the end goal of replacing neurons lost due to damage or neurodegenerative disease would be invaluable. Below we discuss the burgeoning field devoted to directed differentiation of neurons.

### Glia to Neuron Reprogramming

The reasoning behind using cortical glia as substrates for reprogramming to neurons stems from several characteristics of glial cells. Glial cells are plentiful in the cortex (Azevedo et al., 2009) and are actively generated throughout life (Colodner et al., 2005; Geha et al., 2010; Yeung et al., 2014). Therefore, a lack of substrate cells is not an issue. Furthermore, in response to injury and disease, glial cells become reactive and increasingly proliferative, allowing for an increased number of cells to reprogram at the injury site.

One of the first studies that investigated the ability of single transcription factors to induce directed differentiation of cortical astroglia to specific neuronal subtypes was done by isolating cortical glia from postnatal day 5–7 mice followed by retrovirus-induced expression of *Neurog2* or *Dlx2* (Heinrich et al., 2010). As mentioned above, *Neurog2* is a proneural gene that regulates the early differentiation of glutamatergic neurons (Hevner et al., 2006; Heng et al., 2008; Zhang et al., 2013). In contrast, the *Dlx2* homeobox protein is normally expressed in progenitor cells derived from the ventral telencephalon and has been shown to promote the generation of GABAergic interneurons (Petryniak et al., 2007; de Chevigny et al., 2012). Expression of *Neurog2* in astroglial cultures led to the generation of glutamatergic-like neurons which expressed Tbr1 and Tbr2, the T-box transcription factors expressed by glutamatergic neurons in the forebrain. The *Neurog2*-induced neurons also generated synapses, expressed vGluT1 around their soma and MAP2^+^ processes, and acquired projection neuron-like morphology. In contrast, the *Dlx2-*induced neurons expressed markers of GABAergic interneuron lineage GAD67 and vGAT, and generated functional synapses. Interestingly, the *Dlx2*-mediated reprogramming was not as efficient as *Neurog2*-induced reprogramming.

The majority of glia to neuron conversion studies in the brain have been reported in regions other than the cortex. Several groups have reported successful conversion of glia to neurons in the striatum by the expression (or knockout) of various factors (Niu et al., 2013; Torper et al., 2013; Magnusson et al., 2014). The expression of *Brn2a*, *Ascl1*, and *Mytl1* (also known as BAM factors) was previously shown to convert fibroblasts and hepatocytes to neurons *in vitro* (Vierbuchen et al., 2010; Marro et al., 2011). Expression of the BAM-factors mediated by a Cre-inducible lentiviral injection into the striatum of GFAP-Cre heterozygous mice converted striatal glia to neurons. These induced neurons expressed NeuN and had neuronal morphology; however the functionality of the induced neurons was not assessed (Torper et al., 2013).

Notch signaling is involved in various stages of cortical neurogenesis and generally functions to inhibit neuronal differentiation (Ables et al., 2011). A recent study has shown that an experimental model of middle cerebral artery occlusion-induced stroke results in transient neurogenesis in striatal astrocytes (Magnusson et al., 2014). Notch1 signaling was reduced in striatal astrocytes after stroke while ectopic activation in astrocytes inhibited stroke-induced neuroblast production. Furthermore, blocking notch signaling in the absence of stroke promoted astrocytes in the striatum and medial cortex to enter a neurogenic program and express markers of immature and mature neurons, such as Ascl1, Dcx, and NeuN (Magnusson et al., 2014). Taken together, this suggests that Notch signaling actively suppresses the neurogenic potential of parenchymal astrocytes in the striatum (Figure 2B). Lastly, several studies have shown that single factors can convert glia to neurons after injury (Guo et al., 2014; Heinrich et al., 2014). These are discussed below.

### Cortical Neurogenesis after Inflammation and Injury

Over the last decade, plentiful information has been discovered about the relationship between inflammation and neurodegenerative disease. Previous dogma understood neuroinflammation to always have a negative role in disease progression. However, recent studies have shown that certain aspects of the immune system are beneficial to the host in neurodegenerative conditions such as amyotrophic lateral sclerosis (ALS), Alzheimer’s disease and multiple sclerosis (McCombe and Henderson, 2011; Weitz and Town, 2012; Breunig et al., 2013; Guillot-Sestier and Town, 2013; Hussain et al., 2014). In respect to cortical neurogenesis, neuroinflammation is a double-edged sword. Numerous groups have reported that inflammation promotes cortical neurogenesis and conversion of various cell types to neurons in the cortex (Guo et al., 2014; Heinrich et al., 2014; Magnusson et al., 2014). As mentioned above, several groups reported that laser-induced injury and apoptosis in deep layers of the cortex can stimulate precursors *in situ* to increase division and differentiation into glutamatergic neurons (Magavi et al., 2000; Brill et al., 2009). However, a follow up study which used a neuronal promoter-driven caspase as well as a neuronal promoter-driven diphtheria toxin method of induced apoptosis reported increased proliferation of microglia, but did not promote generation of glutamatergic neurons (Diaz et al., 2013). In respect to transgene-induced conversion, the retrovirus-mediated misexpression of *Sox2* in the adult mouse cortex following stab wound injury induced the conversion of NG2 cells to interneuron-like cells (Figure 2B), whereas misexpression under normal (non-stab wound) conditions did not result in conversion (Heinrich et al., 2014). The resulting induced neurons exhibited voltage-and time-dependent conductance and received synaptic connections from endogenous GABAergic neurons. This study used NG2 glia cells as a substrate, a cell type that is abundant and proliferating in the adult brain (Dimou and Gotz, 2014). Other studies have shown that NG2 cells are recruited to the site of injury and may be equally or more permissive to conversion than astrocytes, suggesting that future reprogramming efforts should not look over this cell type (Buffo et al., 2005, 2008; Hughes et al., 2013). Interestingly, (Buffo et al., 2005) reported that stab wound injury increased expression of *Olig2* by immunohistochemistry and mRNA expression, and 26% of these *Olig2*^+^ cells were NG2 cells. Furthermore, increased *Olig2* expression after stab wound was accompanied by decreased *Pax6* expression. This *Pax6*-*Olig2* relationship is also seen in the developing spinal cord and subependymal zone (Mizuguchi et al., 2001; Hack et al., 2005). Inhibition of *Olig2* by retrovirus encoding a dominant-negative form of *Olig2* two days after stab wound resulted in increased *Pax6* expression and increased neurogenesis as evidenced by the generation of Dcx^+^ neuroblasts. The retroviral-mediated overexpression of *Pax6* two days after stab wound injury also resulted in similar increases in *Pax6* expression and neurogenesis.

It was also reported that retroviral expression of *NeuroD1* in the cortex of adult stab wound-injured mice resulted in conversion of: (1) astrocytes to glutamatergic neurons; and (2) NG2 cells to GABAergic and glutamatergic neurons (Figure 2B; Guo et al., 2014). Electrophysiology performed on slice cultures revealed that the *NeuroD1*-converted neurons exhibited spontaneous and evoked synaptic responses. Similar results were seen in the cortex of Alzheimer’s disease mice, but not in control (un-injured or non-diseased) mice (Guo et al., 2014). Similar work was done *in vitro* where adult cortical astrocytes where isolated after stab wound injury and converted to glutamatergic neurons by *Neurog2* or GABAergic interneurons by *Dlx2* as previously discussed (Heinrich et al., 2010). Taken together, these reports suggest the importance of the neuroinflammatory response for conversion to neuronal subtypes in the adult mouse cortex under certain conditions.

Of increased interest in the Heinrich et al. (2014) and Guo et al. (2014) reports, is the idea that transcription factors can exhibit opposing roles depending on the context. Developmentally, *Sox2* promotes self-renewal in neural stem cells and prevents their differentiation into neurons (Graham et al., 2003). However, when *Sox2* is expressed in NG2 cells after injury, it promotes their reprogramming to interneuron-like cells as described above Heinrich et al. (2014). A similar study in the adult mouse striatum reported the conversion of astrocytes to other neuronal subtypes upon induction of *Sox2* (Niu et al., 2013). The mechanism of these differential responses remains to be determined. It may reflect different expression levels of the transgene, the epigenetic state of the transduced cells, or extrinsic cues associated with the neuroinflammatory state of the substrate cells and their microenvironment. Going forward, an integrated analysis of genome-wide binding sites, interacting proteins (e.g., other transcription factors and co-factors), chromatin configuration, and other epigenetic marks in these varied spatiotemporal contexts will likely illuminate the disparate responses to transcription factor misexpression (Amador-Arjona et al., 2015).

Interestingly, the majority of *in vivo* glia-to-neuron conversion studies have been carried out in an inflamed neocortex. Importantly, the activation of the immune system in response to injury or disease usually results in perpetuated and chronic neuroinflammation, which eventually leads to neurotoxicity. The glial scar is an example of this as the resulting activated astrocytes after injury are initially beneficial but eventually form a chronic scar which serves as a barrier to neuronal regeneration (Sofroniew, 2009; Cregg et al., 2014). In respect to the examples discussed above regarding expression of *Sox2* o*r NeuroD1* in NG2 cells (Guo et al., 2014; Heinrich et al., 2014), future studies will need to determine what exactly is different in the NG2 cells or their environment after injury that allows for their reprogramming. Is it only that they are cycling more rapidly? Is the inflammatory microenvironment transiently more permissive to reprogramming in the cortex? And if so, can these features be mimicked in the un-inflamed cortex for a more translatable approach to replacing cortical neurons? Indeed there is some indication that activation of innate immunity is necessary for reprogramming. Specifically, it was noted that TLR3 pathway activation induced notable epigenetic changes leading to chromatin alterations which enhanced the pluripotent stem cell conversion of fibroblasts by four reprogramming factors (Li et al., 2009, c-Myc; Lee et al., 2012). When TLR3 (or its adaptor protein TRIF) was knocked down, reprogramming efficiencies decreased. Interestingly, the authors noted that conventional retroviral methods of transgene expression activate the TLR3 pathway, and non-viral methods of transgene expression complemented with ectopic TLR3 activation increased reprogramming efficiencies. Specifically, a change in the methylation status of the *Oct4* and *Sox2* promoters was observed. These authors claim that there is an optimal window of immune activation necessary for reprogramming (Cooke et al., 2014). They term this the “Goldilocks zone.” A greater understanding of such a phenomenon in the context of neuronal reprogramming might yield more powerful control over the process. In this regard, it is also possible that the neuroinflammatory state present in the successful neuronal reprogramming experiments discussed above involve an activated TLR3 pathway resulting in an open, “pro-reprogramming” chromatin configuration.

### Other Cell Types

Reprograming strategies in cell types other than neurons and glia may also show promise for cortical neurogenesis. (Brill et al., 2009) isolated human pericytes from the adult cerebral cortex and converted them to neurons by the misexpression of *Ascl1* and *Sox2*. Pericytes are involved in regulation of blood flow in the brain and the establishment and maintenance of the blood brain barrier (Armulik et al., 2011). Interestingly, they have also been reported to be multipotent mesenchymal stem cell-like and can give rise to cartilage, muscle, and bone lineages (Armulik et al., 2011). Since these cells are dispersed throughout the cortex and are more abundant than neural stem cells, they may be an attractive substrate for *in vivo* reprogramming to cortical neurons.

### Neuron to Neuron Reprogramming

While the above studies provide important insight into the phenomenon of neuronal reprogramming, they all employ transcription factors broadly expressed in diverse types of neurons (Table 1). And while it is clear that neurons are generated, detailed characterization awaits. Given these facts, the precise nature of the resulting reprogrammed populations are hard to predict and may be extremely heterogeneous. For example, this approach might yield the types of populations seen after differentiation of pluripotent cell types, namely many neurons exhibiting “stalled” phenotypes and aberrant expression of phenotypic markers (Sadegh and Macklis, 2014). Going forward, it may be necessary to employ additional specific factors to direct the differentiation of precise subtypes. One such example of a protein which may be utilized is the transcription factor Fezf2. During embryogenesis, *Fezf2* is necessary for the specification of corticospinal motor neurons and *Fezf2*^−/–^ mice lack corticospinal motor neurons (Chen et al., 2005a, b; Molyneaux et al., 2005). Embryonically, forced expression of *Fezf2* is sufficient to reprogram progenitors destined to generate upper layer neurons or striatal neurons to corticospinal motor neurons (Chen et al., 2005b; Molyneaux et al., 2005; Rouaux and Arlotta, 2010). Elegant work by Rouaux and Arlotta (2013) has shown that early postnatal overexpression of *Fezf2* in postmitotic Layer 2/3 callosal projection neurons which typically project interhemispherically via the corpus callosum, results in reprogramming to Layer 5-like corticofugal neurons (Figure 2B). The newly generated neurons acquired the molecular properties of Layer 5 neurons expressing markers ER81 and CRYM, and downregulating CUX1. Axonal projections in the newly reprogrammed cells were also re-directed from interhemispherical targets to subcortical targets. (De la Rossa et al., 2013) has shown that early postnatal overexpression of *Fezf2* in Layer 4 spiny neurons changes their identity to Layer 5-like corticofugal neurons. Taken together, these two reports indicate that postmitotic neurons, which are already in their respective cortical layer and have defined their projections, still maintain a sense of plasticity to reprogramming catalyzed by single transcription factor overexpression in early postnatal life. However, both groups reported decreased reprogramming efficiencies at postnatal day 21, suggesting that the plasticity of these postmitotic neurons declines with age and will likely be absent in the adult brain. Interestingly, when *Fezf2* is expressed in SVZ progenitors destined to become OB GABAergic interneurons, it directs fate towards a glutamatergic phenotype (Zuccotti et al., 2014). The resulting *Fezf2*-respecified OB neurons have features akin to pyramidal cells including larger cell bodies, elaborative dendritic trees, and pyramidal neuron-like electrophysiological outputs. However, the reprogramming reported does not appear to be as complete as is seen in the studies performed embryonically or perinatally. Interestingly, a recent study has also reported high Fezf2 expression in a subset of callosal neurons in the adult cortex, suggesting that Fezf2 expression may not be restricted solely to corticospinal motor neuron fate (Tantirigama et al., 2014). Taken together, the findings of the reprogramming studies discussed above suggest a reduction in reprogramming ability by *Fezf2* with age. Given the complex combinatorial transcriptional co-regulation of neuronal specification during development (Kwan et al., 2012; Greig et al., 2013), this is perhaps not surprising.

**Table 1:** **Reported examples of transcription factor-mediated postnatal cortical neuronal conversion**.

Factor(s)	Gene introduction	Age of animal	Region	Substrate cell	End cell (similar to)	Reference
Fezf2	Electroporation	Neonate	Layer 2/3 cortex	Callosal projection neuron	Corticofugal projection neuron	Rouaux and Arlotta (2013)
Fezf2	lontoporation	Neonate	Layer 4 cortex	Spiny neuron	Corticofugal projection neuron	De la Rossa et al. (2013)
Fezf2	Lentivirus	Neonate/Adult	Lateral ventricle	SVZ progenitor	Glutamatergic pyrimidal	Zuccotti et al. (2014)
					Neuron in OB	
RBP-Jk	Conditional KO	Adult	Medial cortex	Astrocyte	Dcx+ neuron	Magnusson et al. (2014)
dn-Olig2	Retrovirus	Adult	Stab wound	Olig2+ cell	Dcx+ neuron	Buffo et al. (2005)
			injured cortex			
Sox2	Retrovirus	Adult	Stab wound	NG2 cell	Dcx+ neuron	Heinrich et al. (2014)
			injured cortex			
Neurod1	Retrovirus	Adult	Stab wound	Astrocytes	Glutamatergic neuron	Guo et al. (2014)
			injured cortex			
Neurod1	Retrovirus	Adult	Stab wound	NG2 cell	Glutamatergic and	Guo et al. (2014)
			injured cortex		GABAergic neurons	

Furthermore, as suggested by various examples discussed above, a single transcription factor may have several respective roles and its expression may result in different outcomes depending on the time and context of its expression. For example, *Sox2* promotes self-renewal in neural stem cells and is one of the Yamanaka factors for induced pluripotency (Graham et al., 2003). However, when *Sox2* is expressed in NG2 cells after stab-wound injury or in adult astrocytes in the striatum, these cells can be reprogrammed to interneuron-like cells (Heinrich et al., 2014) or neuroblasts respectively (Niu et al., 2013). These results suggest the need to carefully sculpt strategies for directed differentiation by incorporating developmental logic, knowledge of the donor cell properties, knowledge of the host tissue, and forethought in avoiding situations which might be deleterious clinically (e.g., tumor growth due to transgene misexpression; Nori et al., 2015). Employing such developmental metrics combined with precise tools for genetic manipulation will likely be increasingly necessary in bolstering reprogramming efficiencies to generate precise neuronal subtypes (Victor et al., 2014; Akhtar et al., 2015). Lastly, the use of accurate disease-specific models systems and transplant hosts—including aged genetically modified rodents, non-human primates, and potentially human organoids—will likely be of the utmost importance to assess the translational capacity of potential novel therapies.

## Conclusions

Grafting and transplantation of neuronal tissue has proceeded for decades with little or no progress towards clinical therapies for cortical trauma and degeneration. However, a critical mass of findings in multiple subfields promises to improve this situation. The development of iPSC technology has re-formatted our conception of cell differentiation. Moreover, this technology provides virtually unlimited “starting material” for the generation of cells for transplant. Our knowledge of cortical development has greatly informed our ability to rationally direct differentiation of neurons from pluripotent stem cells. Specifically, employing developmental paradigms to direct neuron subtype differentiation holds the promise of enabling mix and match generation of neurons lost to injury and disease. Nevertheless, as we have detailed, meaningful clinical replacement of cortical circuitry will be among the most difficult problems for modern science to solve. Generating the appropriate cell types will only partially solve this challenge. The next step is to bridge the gap between the precise specificity of circuits created during development with our ability to transplant or reprogram cell types in a manner that allows for the recapitulation of these circuits. With a trillion synapses per centimeter of lost tissue, this will be no small feat. However, given the lack of alternatives, we have no choice but to “beat on, boats against the current,” in attempting to rise to the challenge of creating efficacious clinical treatments for those who would otherwise suffer lifelong disability.

## Conflict of Interest Statement

The authors declare that the research was conducted in the absence of any commercial or financial relationships that could be construed as a potential conflict of interest.
